# Mental Distress, Help Seeking, and Use of Health Services Among University Students. The SHoT-Study 2018, Norway

**DOI:** 10.3389/fpsyt.2021.727237

**Published:** 2021-10-20

**Authors:** Marie Husøy Sæther, Børge Sivertsen, Ottar Bjerkeset

**Affiliations:** ^1^Faculty of Nursing and Health Sciences, Nord University, Levanger, Norway; ^2^Department of Health Promotion, Norwegian Institute of Public Health, Bergen, Norway; ^3^Department of Research & Innovation, Helse Fonna HF, Haugesund, Norway; ^4^Department of Mental Health, Faculty of Medicine and Health Sciences, Norwegian University of Science and Technology, Trondheim, Norway

**Keywords:** higher education, mental health, health services, help-seeking, student subgroups

## Abstract

**Background:** Existing studies have documented high levels of mental distress in University and college students, complemented with poor help-seeking behavior. Colleges and universities offer a unique setting to address mental health problems that might overcome some of the most prominent barriers to help-seeking.

**Objective:** We aim to describe the use of campus-based health care services and health services available in the near-by community among students in Norwegian student welfare organizations. We compare health care service use between non-local (in-movers) and local students, students at large and small welfare organizations, and students with severe and medium-low levels of mental distress.

**Methods:** Data stem from the SHoT study (Students' Health and Well-being Study), a national survey from 2018 of all students aged 18–35 undertaking higher education in Norway. Mental distress was assessed using the Hopkins Symptom Checklist-25 (HSCL-25), and we also obtained self-report data on use of health care services. Data on health care services offered at Norwegian student welfare organizations was obtained from semi-structural telephone interviews.

**Results:** Non-local students used health care services that are low threshold, easily accessible and close to campus (health clinics and services organized by the student welfare organization) to a larger extent than local students. Students with symptoms of severe mental distress used almost all types of health services more than other students. We found big differences in reported use of health services in large and small organizations, yet these differences mirrored services available, and not necessarily student demand and preferences.

**Conclusion:** Services offered by the student welfare organizations seem to play a particularly important role for non-local students and students reporting symptoms of severe mental distress.

## Introduction

The high and increasing prevalence of mental distress and health problems in University students have become cause for concern. Cross-national studies carried out by the WHO suggest that about one third of college students have experienced mental health problems in the past 12 months (1, 2), and there are strong indications of a sharp increase in the prevalence among youth and students in general over the last decade (3, 4). For some, the period of entering higher education is a time associated with increased personal and academic stressors. In addition, students are also a high-risk population due to their age, as most lifetime mental disorders have their onset before 25 years of age (5, 6). For vulnerable students, the encounter with University life may therefore lead to, or worsen already existing mental health problems (7).

Surveys among students at European universities and colleges indicate that the global prevalence of mental health problems suggested by the World Health Organization (WHO) is representative of the situation in Europe, Scandinavia and Norway. Studies have been conducted at campuses in several countries, including the UK (7–9), Turkey (10), Spain (11), Norway (12), Germany (13), France (14), and Sweden (15, 16). Most of these studies report a prevalence of moderate to severe self-reported mental health problems as high as 30%. Most students impaired by this suffer from symptoms indicative of depression and anxiety (17, 18). However, findings from existing studies also indicate that these students are reluctant to seek help and many do not receive adequate treatment (19). Many prefer to handle mental health challenges alone or by support from close friends and relatives (20). Further, many students experience substantial barriers to help-seeking, including fear of stigma, lack of trust in services, and costs (21).

Scholars and health workers who have studied the student population over time, state that there is an urgent need to counter the trend of increasing mental health problems among students (22, 23). By now, there is a growing awareness of the importance of school mental health as an aspect of health promotion (24), leading national governments and international organizations (e.g., WHO) to put children and youth's mental health on their agendas and to encourage investments in infrastructures that promote mental health in students, such as school- and University-based health services or web-based programs (25).

Colleges and universities offer a unique setting to address mental health problems among young adults and attend to them before they grow in severity and cause long-term problems. All students at Norwegian universities and colleges belong to a student welfare organization that offers a variety of services, including advisory and health care services, sports facilities, housing, and kindergartens. The membership is mandatory for all students and the organizations are, by regulation, obliged to ensure that students have access to all types of welfare services needed given the study-specific context (26). For the most part, this means supplementing local public and private services rather than supplying full-scale health care services. More specifically, to make sure students have easy access to low-threshold and free-of-charge or subsidized services to consult in the case of less urgent health problems, such as mild to moderate mental distress, testing for sexually transmitted diseases (STDs) or prescriptions of contraceptives. This kind of availability and the image of supplying low-threshold services might create the opportunity for student welfare organizations to reach students and young adults in a way than cannot be matched by other institutions, and to overcome some of the barriers to help-seeking, including e.g., fear of stigma, financial constraints, lack of information about help and mental health services available, and time constraints (27, 28).

The regulation defining the purpose of student welfare organizations does not include a detailed description of services to be offered by the organization, other than its responsibility to ensure that students have access to the welfare services they are in need of. Hence, depending on campus size and location, the student welfare organization can to various degrees rely on existing services supplied by the local community. As a result, there is reason to believe that the health care services currently supplied by the student welfare organization vary considerably between organizations and campuses. If students experience on-campus health services as easily accessible and low-threshold compared with services in the local community, such variation might translate into differences in help-seeking and treatment. In particular, this might affect local and non-local students differently, due to differences in social network and information about the local services.

The aim of this study is to describe the use of health care services among students at Norwegian universities and colleges. Using student self-report data from The Students' Health and Well-being Study 2018 [Studentenes Helse- og Trivselsundersøkelse] (the SHoT 2018-study), we describe the prevalence of symptoms of mental health distress and help-seeking patterns in relation to gender, symptom severity, among local and non-local students and students from large vs. small Norwegian student welfare organizations. To complement the information on student health care service use, information about the supply of on-campus health care services is collected through semi-structured interviews with representatives from Norwegian student welfare organizations.

## Materials and Methods

### Setting and Participants—The SHoT-Study

The SHoT-study is a Norwegian survey on mental and physical health, quality of life, health related behaviors and medication use among students in higher education, carried out over three waves in 2010, 2014, and 2018. In 2018, an invitation to participate was sent to all Norwegian full-time students (enrolled in Norwegian universities and colleges or studying abroad) in the age group 18–35. The study was conducted between February and April. The participation rate for the 2018-survey was 31% (50,054 students), with higher relative participation for female students compared with male students (4, 29).

#### Variables/Measures of Student Characteristics and Mental Health

All variables related to demographics, self-reported mental health, help-seeking, and use of health care services among students were obtained by from the SHoT-2018 study. The participants were asked about their gender, age and whether they considered themselves new in their study city (non-local students). The SHoT questionnaire included the Hopkins Symptoms Checklist (HSCL-25), a widely used screening instrument for psychological distress, mainly anxiety and depression symptoms (12, 30). It has been used in a variety of health care in general population settings and provide good screening performance and psychometric properties. An investigation of the factor structure based on the SHoT2014 dataset showed that a uni-dimensional model had the best psychometric properties in the student population and not the original subscales of anxiety and depression (31). Cronbach's alpha for the version of HSCL-25 used in the 2018 SHoT-survey is 0.94.

In the HSCL-25 each question represents a claim relating to presence of a symptom of psychological distress and is answered on a 4-item Likert scale from 1 (not at all) to 4 (extremely). The period of reference was the past 2 weeks. An average score above 1.75 is interpreted as an indication of moderate to severe symptoms of mental distress (32, 33), an average score above 2.0 as an indication of severe mental distress (34). The questions from the HSCL-25-list do not (on their own) provide sufficient foundation to make conclusions about an individual's mental health state, level of functioning, a psychiatric diagnosis or quality of life without a clinical evaluation. Finally, the participants were asked to rate their overall/general health (bad, not very good, good, very good).

#### Use of and Satisfaction With Health Services

Students were asked about their use of and satisfaction with health care services. The questions cover overall service use and frequency of use of general practitioner (GP), Out-of-hours GP, health clinic/nurse, and psychologist/psychotherapist, and whether these services were supplied by the local student welfare organization. Student health clinics mainly offer services related to physical and sexual health, such as testing for STDs and prescriptions of contraceptives, but also psychological counseling. Students reporting use of GP or Out-of-hours GP, were asked whether their visits the last 12 months were due to physical or mental health problems (yes/no on both). Satisfaction with health services was rated on a 1–5 scale (very dissatisfied, quite dissatisfied, neither/nor, quite satisfied, very satisfied). In our analyses, we have grouped “very satisfied” and “quite satisfied” into one combined category (satisfied) indicating satisfaction with local health care services.

#### Missing Data

In total, 50,054 students participated in the survey. However, some students did not answer all the questions covering our interest variables. Hence, our sample is therefore slightly smaller. Observations with missing information on student welfare organization membership, gender and age, where none of the 25 questions in the HSCL-25 list were answered, students from the smallest organization (with only 11 observations), and students with gender other than male/female were dropped from the sample from the SHoT-data. In total, this summed up to 2,576 students, leaving us with a sample of 47,478 student observations (94.9% of the total number of respondents of 50,054) from 13 out of 14 Norwegian student welfare organizations. Further, some students did not answer the survey question asking whether they had moved to a new area when enrolling in higher education. Hence, in analyses comparing local and non-local students (students that had to move from their hometown to the University town/students considering themselves new to the city), the final sample consisted of 47,362 student observations (94.4% of the total number of respondents).

### Provision of Health Care Services Within Student Welfare Organizations

Information about health care services offered by the student welfare organizations during the academic year 2017–2018 was obtained by the first author (MHS) from semi-structured telephone interviews with representatives from each organization, supplemented with information from the organizations' websites (one of the organizations was not available for interview). The interviews were conducted between December 11th 2020 and March 1st 2021. The interviews comprised questions on types of health care services offered by the student welfare organization, collaborations with local providers of health care, availability (location and waiting lists), and financing and user costs. Health services offered within student welfare organizations associations are free of change or heavily subsidized. Student welfare organizations were defined as either small (<20,000 students) or large (>40,000 students). All welfare associations could be classified according to these groups as none of them had between 20,000 and 40,000 members. Three out of four students were members of a large student welfare organizations. The small organizations had between 4,000 and 20,000 members. The size of the student welfare organizations mirrors the size of the universities and colleges located in the respective geographical area. Some student welfare organizations cover a very wide geographical area. The longest distance between two campuses within one organization amounts to over 800 km (the case in two organizations).

### Statistical Analysis

Multivariate logistic regressions were used to estimate associations between binary outcomes indicating symptom pressure of mental distress or use of health care services and student characteristics (local vs. non-local students, students at large student welfare organizations vs. small organizations, and students with high vs. moderate-low levels of self-reported mental distress). All regressions were adjusted for age and sex, and standard errors were clustered at the student welfare organization level. All analyses were run using the Stata MP 15.1 software.

## Results

### Student Characteristics and Use of Health Care Services in SHoT 2018

Descriptive statistics on demographics, mental health and use of health care services for SHoT-2018 participants are shown in Table 1. The sample used in the below analyses consisted of 47,478 students. The proportion of female students was 69%, and there were about equal shares of students aged 18–22 and 23–35. Two out of three students had moved to a new area or city when entering college or University, and the same proportion of students belonged to a large welfare organization.

**Table 1:** Descriptive statistics: student characteristics, mental health and use of health care services: *N* = 47,478.

	**Total**	**Female**	**Male**
**Demographics**			
Female	69.2%		
Age 18–22	49.4%		
Age 23–35	50.6%		
Moved to a new city to enter higher education	67.0%^a^	66.0%	69.1%
Belong to a large student welfare organization (#students>20,000)	66.8%	66.8%	66.9%
**Symptoms of mental distress**			
Average HSCL-25-score (cont. range 1–4) (standard deviation)	1.73 (0.549)	1.82 (0.555)	1.53 (0.481)
Average HSCL-25-score>2	26.5%	31.4%	15.5%
**Use of health care services**			
Visited GP	59.4%	64.8%	47.2%
Use of out-of-hours GP	12.6%	13.7%	10.2%
For physical problems (GP/ER last 12 months)	40.4%	45.3%	29.2%
For mental problems (GP/ER last 12 months)	15.0%	17.6%	9.0%
Use of health clinic/nurse	13.9%	17.7%	5.3%
Help-seeking at psychologist/psychotherapist	10.4%	11.9%	7.0%
**Satisfaction with local health care services** ^b^	64.9%	66.0%	62.5%

a *N = 47,362*;

b *Includes both answers quite satisfied and very satisfied with local health care services*.

The average score on the HSCL-25 questionnaire was 1.73, with 26.5% of the students having an average score higher than 2, indicting a severe symptom load. Reported symptoms of mental distress were notably higher in female than in male students. The average HSCL-25-scores were 1.82 and 1.53 and the share of students with an average score higher than 2 were 31.4 and 15.5%, for female and male students, respectively.

Overall, about 60% of students had visited a GP and 13% had visited Out-of-hours GP located in the study city/region during the last 12 months. Forty percentage of the sample gave physical health problems and 15% reported mental health problems as the main reason for their visit(s) to the GP, Out-of-hours GP or emergency room at hospital (ER). A total of 6,592 students (13.9%) reported to have consulted a health clinic or a nurse. Of these, only about 9% reported monthly (or more frequent) visits. Further, 4,937 students (10.4%) had been to a psychologist/ psychotherapist, three out of four of these at least as frequently as once a month. All help-seeking patterns, but particularly use of health clinic/nurse and mental health services, were more common in female than male students explain. Two thirds of students reported to be quite or very satisfied with the local health care services, a slightly higher proportion in females compared to males (66.0 vs. 62.5%).

Table 2 reports differences in use of health care services between non-local and local students, and between students in large and small student welfare organizations. Overall, the prevalence of self-reported symptoms of mental distress was similar in local and non-local students (OR = 0.95, 95% CI 0.88–1.02), and across students in large and small student welfare organizations (0.90, 95% CI 0.75–1.08).

**Table 2:** Binary associations between use of health services in non-local (*N* = 31,714) vs. local students (*N* = 15,648) and students belonging to large (*N* = 31,722) vs. small (*N* = 15,756) student welfare organizations, presented as odds ratios.

	**Non-local students**		**Student in large welfare organizations** ^**b**^	
	**(vs. local students)** ^**a**^		**(vs. small welfare organizations)**	
	**OR**	**95% CI**	**OR**	**95% CI**
Average HSCL-25-score>2	0.95	(0.88–1.02)	0.90	(0.75–1.08)
**Use of health care services**				
Visited GP	0.80	(0.67–0.95)	1.21	(1.01–1.45)
Use of out-of-hours GP	0.94	(0.89–0.99)	0.76	(0.63–0.92)
For physical problems (GP/ER last 12 months)	1.02	(0.95–1.09)	1.20	(1.04–1.39)
For mental problems (GP/ER last 12 months)	1.05	(1.00–1.10)	1.09	(0.96–1.26)
Health clinic/nurse	1.58	(1.39–1.80)	0.66	(0.45–0.96)
Psychologist or other therapist	1.07	(1.03–1.12)	1.27	(1.10–1.47)
**Use of student welfare organization health services**				
Visited GP	3.12	(2.33–4.18)	19.21	(8.35–44.17)
Health clinic/nurse	2.56	(2.14–3.06)	0.69	(0.30–1.54)
Psychologist/psychotherapist	1.61	(1.48–1.75)	1.58	(1.00–2.50)
**Satisfied with local health care services**	1.09	(0.99–1.21)	2.01	(1.57–2.55)

*Adjusted for sex and age, age included as continuous variable. Standard errors clustered at the student welfare organization level*.

a *N = 47,362*.

b *Student welfare organizations classified as large include the three largest in Norway*.

#### Health Service Use in Non-local vs. Local Students

Non-local students were less likely to visit a GP or Out-of-hours GP in the community where they study, compared with local students (OR = 0.80, 95% CI 0.67–0.95, and OR = 0.94, 95% CI 0.89–0.99) (Table 2). Among those using these services, the reason for their visits (mental or physical health problems) did not differ between local and non-local students. The proportion of students that had visited a health clinic/nurse was about 60% higher, and use of psychotherapy 7% higher, among non-local compared to local students.

Non-local students used all services offered by the student welfare organization notably more than local students; they were about 2–3 times more likely to visit the health clinic/nurse and GP, and about 60% more likely to see a psychologist/psychotherapist when services were offered within the welfare organization, compared to local students.

#### Health Service Use in Large vs. Small Student Welfare Organizations

Students in large student welfare organizations had a 20% increased odds of using GPs in general and a 19-fold increased odds of having used GPs working for the student welfare organizations, compared to students from small welfare organizations.

The use of health clinics was lower among students belonging to large student welfare organizations (OR = 0.66, 95% CI 0.45–0.96), while the use of psychologist/psychotherapist was more frequent among students in large organizations (OR = 1.27, 95% CI 1.10–1.47) compared to small. Looking at the use of mental health services supplied by the student welfare organizations, students at large organizations were about 60% more likely to visit psychologist/ psychotherapist than students in small organizations.

#### Satisfaction With Health Services

Table 2 shows that twice as many students in large welfare organizations were satisfied with their health services offered where they study, compared to small welfare organization students. Although statistical evidence was borderline, non-local students seemed slightly more satisfied with the services compared to local students (OR = 1.09, 95% CI 0.99–1.21).

#### Health Care Use in Students With High vs. Low-Moderate Symptom Load

Students with symptoms of severe mental distress (average HSCL-25-score>2) used all types of health care services more frequently than other students, except for health clinics/nurses (Table 3). In particular, use of psychologist/psychotherapist, and GP, Out-of-hours GP, or ER due to mental health problems, was notably higher in this sub sample of students: Students with an average HSCL-25-score above 2 were 4–5 times more likely to use these services than other students, and 3 times more likely to receive mental health treatment (psychologist/ psychotherapist) at their student welfare organizations. However, this group was less satisfied with health services than those with lower levels of psychological distress (OR = 0.62, 95% CI 0.60–0.64). Among students with an average HSCL-25-score higher than 2, about 50% reported to be in good health or in very good health.

**Table 3:** Binary associations between use of health services in students with high symptom load (avg. HSCL-25-score>2) (*N* = 12,576) vs. students with low-moderate symptom load of mental distress (avg. HSCL-25-score ≤ 2) (*N* = 34,902), presented as odds ratios.

	**Students with high vs. low-moderate symptom load of mental distress**	
	**OR**	**95% CI**
**Use of health care services**		
Visited GP	1.23	(1.19–1.28)
Use of out-of-hours GP	1.28	(1.22–1.36)
For physical problems (GP/ER last 12 months)	1.38	(1.33–1.44)
For mental problems (GP/ER last 12 months)	4.39	(4.10–4.69)
Health clinic/nurse	0.83	(0.76–0.89)
Psychologist or other therapist	5.00	(4.47–5.58)
**Use of student welfare organization health services**		
Visited GP	1.12	(0.99–1.26)
Health clinic/nurse	0.81	(0.72–0.90)
Psychologist/psychotherapist	3.01	(2.71–3.35)
**Satisfied with local health care services**	0.62	(0.60–0.64)

*Adjusted for sex and age, age included as continuous variable. Standard errors clustered at the student welfare organization level*.

### Student Welfare Organization Health Care Services

Table 4 summarizes the supply of health care services in Norwegian student welfare organizations. Some differences in the supply of services across campuses (within the organization) exist; however, most organizations manage to offer a similar portfolio of services to all their students. Hence, differences in services available to students are a feature mainly between organizations, and not within. All Norwegian student welfare organizations had counselors offering advisory services for students seeking help for mild to moderate mental health problems. For three of the organizations, this was the *only* type of health service offered (not displayed in the table). The counselors were health workers, but not licensed therapists, such as psychologists or psychotherapists.

**Table 4:** Supply of health care services in small and large student welfare organizations.

	**Student welfare organization size**	
	**Large (>40,000 students) *N* = 3**	**Small (≤20,000 students) *N* = 10**
**Supplied within the student welfare organization**		
Counselor	3/3 100%	10/10 100%
Nurse/health clinic	1/3 33%	5/10 50%
Psychologist/psychotherapist	3/3 100%	6/10 60%
GP	1/3 33%	0/10 0%
Dentist	2/3 67%	0/10 0%
**Supplied in the local region and made accessible through the student welfare organization**		
Nurse/health clinic	2/3 67%	6/10 60%
Psychologist/psychotherapist	0/3 0%	2/10 20%
GP	2/3 67%	0/10 0%

The three organizations classified as large had psychologists/psychotherapists on staff and offered mental health care services in addition to advisory services. This was only the case in 60% of the small organizations. Two of the small organizations offered mental health services (psychologist) in collaboration with the local community. Hence, three of the organizations classified as small had no mental health care for their students, internally or in collaboration with the local community.

#### Physical and Sexual Health Care Services

Health clinics/nurses were offered more frequently at small student welfare organizations, compared with large. However, in many organizations these services were made available in collaboration with the local community, leaving little difference in the actual supply between organizations and campuses. In many cases, municipality health clinics were perceived as integrated in the health care services provided by the student welfare organization due to a convenient location close to campus. Physical health services (GP and dental services) were only supplied at the large organizations, internally (in one case for GP, in two cases for dentist) or by private clinics located on campus (in two cases for GP).

## Discussion

### Main Findings

In this large national study of more than 47,000 higher education students (SHoT, Norway) in 2018, levels of self-reported symptoms of mental distress and satisfaction with health services were similar in local and non-local students, and across student groups belonging to large and small student welfare organizations. Further, our results demonstrate how important low-threshold and easily accessible services provided by the student organizations are, particularly for non-local students. Students with high levels of anxiety and depression reported to use a wide range of health services, both on campus and community/hospital based, including Out-of-hours GP, and in particular, mental health care services. Further, we found evidence suggesting that differences in health care service preferences between students from large and small student welfare organizations. However, interviews with the organizations revealed that this simply mirrored the available services at the respective campuses and was not necessarily an indication of differences in student demand and preferences.

The share of female respondents has increased from 58 to 59% in the 2010- and 2014-waves of the study to 69% in the current sample. The 2010 and 2018 samples were similar with respect to the age distribution, with equal shares of respondents aged 18–22 and 23–35 years, while the 2014 sample had a slightly older student sample with 38% aged 18–22. The share of students moving to a new area at University or college enrollment was stable over all three surveys (34, 35).

### Levels of Mental Distress in the Student Population

As reported previously from the SHoT 2018 cohort, one in four students had an average HSCL-25-score above 2, indicating severe mental distress (12). This is in accordance with the prevalence presented in previous studies of similar populations (18, 36). Compared with the previous waves of the SHoT-survey from 2010 to 2014, this represents an increase in prevalence. In 2010 16% of students had an average HSCL-25-score higher than 2, in 2014 the prevalence was 21% (4). The increase in the share of students experiencing symptoms of severe mental distress between 2010 and 2018 is found to be stronger in female than in male students (4). To add to the picture, we compared the HSCL-25-score with self-assessed global health. Among students with an average HSCL-25-score higher than 2, about 50% reported to be in good health or in very good health. Hence, the average HSCL-25-score at the chosen cut-off level does only to some extent mirror global health.

### Use of Health Care Services in Different Student Populations

Among the most well-known barriers to mental health help seeking are: male gender, stigma, poor availability and associated costs (21, 37). Our data confirmed the established gender ratio for help seeking, particularly for mental health problems. Previous studies on help-seeking among students suffering from mental distress have found that a large share do not seek and receive help, even when low-threshold campus-based services are available (28, 38). In contrast, our findings indicate that demand for mental health care was pronounced among these students, particularly non-local students. Although the comparison between large and small student welfare organizations might not be relevant for all countries, it should be of great concern how health care needs in students moving from their hometowns to a new area are met.

The majority–two thirds–of Norwegian students moved from home to a new city to study, a larger proportion than in other comparable western countries (39). We are not aware of any existing studies that have investigated use of health services across local and non-local domestic students. However, the importance of available mental health care for international students has been devoted attention (40). Further, it has been found that students living in campus housing were significantly more likely to use mental health service at their campus (psychotherapy) than students living off campus (41). Though this might show the significance of proximity to services offered or active gatekeeping practice rather than a relatively high demand for counseling among non-local students.

### Mental Distress Symptom Burden and Use of Health Care Services

Use of all mental and physical health services, was markedly higher in those with an average HSCL-25-score above 2, the only exception was a 20% reduction in use of health clinic/nurse. Across health care settings, students with the highest symptom burden sought help from psychologists/therapists and GPs three to five times more often than those with a lower symptom burden. This, however, does not provide the foundation for concluding on the *share* of students with depression and anxiety seeking treatment for mental health problems. Previous studies report that most students with mental health problems do not seek and receive help (41), and that help-seeking patterns vary with types of mental health issues (42). We find that many students with psychological distress use student health care services, though we cannot conclude whether this represents a large share of the whole group of students who needs help.

The fact that students who experience the highest symptom burden and are most dependent on these services are less satisfied then healthier and less frequent users, is unfortunate and has been reported previously (43). Common/possible explanations include long waiting lists, unmet needs in therapy, and often the mental condition itself; depressed people, for example, often experience low energy and motivation, are generally pessimistic about their treatment and future outcomes. Nevertheless, it should always be a goal to reach out and accommodate students who struggle, in a way that call hope, trust, reduce stigma and strengthen adherence to the therapy. Students are particularly susceptible to discontinued follow up and therapy, because student life is full of interruptions; brakes, vacation, and studies abroad being some of them.

### Interviews With Student Welfare Organizations

Student mental health treatment can be defined as integrated (offered by the student welfare organization on campus) or divided treatment (both on campus and in community or specialized health care), and referral to specialized health care outside campus (44). Interviews with Norwegian student welfare organizations showed that services offered in the academic year 2017–2018 varied substantially between organizations. In line with our findings, between-campus differences in use of health care services documented in the US context are also found to be related to services available, and not necessarily demand (41).

Further, the SHoT 2018 questionnaire did not specify whether the students had received individual, group therapy, or both. Personal interviews with employees at the student welfare organizations revealed that group based mental health initiatives where not commonly offered before SHoT 2018 yet have become increasingly implemented and popular among students after 2018. Therefore, we can assume that the majority of students who reported help seeking in SHoT 2018 mainly received individual counseling. Of note, group therapy has generally proven good efficacy and might be particularly useful for students; students are familiar with group situations, both in their social lives, living arrangements and academic group learning and exams (45). Group therapy is not only useful in students with psychiatric disorder or psychopathology, but helps improve general factors like autonomy, socialization, self-esteem, and improved interpersonal skills. Mindfulness has also been widely introduced on-campus. Making groups normative, easily accessible, and visible on campus has the potential to reduce stigma and include non-clinical populations. If successful, this might reduce isolation and hopelessness, which in turn might prevent depression (45). Changes in the health care services provided by student welfare organizations since 2018 are mainly related to expanding of group-based health promoting efforts, such as courses, lectures or events. Ten out of 13 organizations have invested heavily in their group-based services since 2018, an investment driven in large by student demand. Only two organizations have invested their funds solely in expanding their capacity in 1-to-1 therapy.

### Strengths and Weaknesses

The SHoT-study provides a large sample size compared with other existing student mental health and well-being studies and includes a wide range of background variables. Invitations to participate were sent to students at *all* Norwegian universities and student welfare organizations, and the final sample includes at least 400 observations from each organization and more than 1,000 observations from 10 of the 13 organizations included in this sample. However, the response rate is relatively modest (31%), and we have little information on non-participants beyond age– and gender distribution, which may limit the generalizability of the findings.

Our study is based on self-report only, and the HSCL-25-score included in our analysis does not indicate or reflect the total symptom burden or number of students with mental health problems and/or mental disorders. Similarly, use of health services obtained by national register linkage would have provided more accurate estimates of off campus service use. In addition, information on psychotropic drug use could shed further light on students' preferences when it comes to dealing with mental health problems. This is, however, outside the scope of this study and will be covered separately in other studies using the SHoT data set. Finally, it should be kept in mind that the recall period for self-reported symptoms of mental distress was 2 weeks, while the recall period for use of health services was 12 months when reading our results.

The SHoT-survey asks students about their use of GP, nurse/health clinic and psychologist/psychotherapists, but not specifically advisory services with counselors without formal license as therapists. Hence, a prominent part of the student welfare organizations' supply did not have a specific category in the SHoT questionnaire. In some cases, students who have seen counselors might have thought of this as seeking help from a therapist or a nurse, potentially giving rise to measurement errors. In addition, collaboration between local health care services and student welfare organizations is common. Some student welfare organizations provide office space for municipality health clinics or GPs on campus and keep user costs low by subsidizing. Therefore, it is not always clear to the student weather whether services are offered by the student welfare organization, a community or specialized clinic. Hence, local public or private services might appear as run by the student welfare organization, suggesting a potential source of measurement errors in reported use of services.

In conclusion, we argue that this study offers new and relevant results from a large-scale student health and well-being survey, well-suited to inform health promotion, new prevention and treatment models offered by student welfare organizations and municipalities hosting University students. Further, there seems to be an urgent need to discuss the structure, organization and financing of these services across Norwegian universities and campuses. Particularly, the high proportion of non-local students are dependent on well-functioning low-threshold services on campus, offered by student welfare organizations. Finally, more rigorous research and evaluations need to be carried out simultaneously with new student health services and initiatives, both in Norway and globally.

## Data Availability Statement

The data analyzed in this study is subject to the following licenses/restrictions: The datasets for this article are not publicly available because of privacy regulations from the Norwegian Regional Committees for Medical and Health Research Ethics (REC). Approval from REC (https://helseforskning.etikkom.no) is a pre-requirement. Guidelines for access to SHoT data are found at https://www.fhi.no/en/more/access-to-data. Requests to access these datasets should be directed to Børge Sivertsen, borge.sivertsen@fhi.no.

## Ethics Statement

The SHoT 2018 was approved by the Regional Committee for Medical and Health Research Ethics in Western Norway (no. 2017/1176). Electronic informed consent was obtained after complete description of the study to the participants. The patients/participants provided their written informed consent to participate in this study.

## Author Contributions

MS and OB contributed substantially to the conceptualization and design of the study. BS was the main investigator and responsible for data collection, data storage, and ethical approval in SHoT 2018. MS performed the statistical analysis and wrote the first draft of the manuscript. BS and OB revised it critically. All authors read and approved the final version of the manuscript and contributed to the interpretation of the data.

## Funding

SHoT 2018 has received funding from the Norwegian Ministry of Education and Research (2017) and the Norwegian Ministry of Health and Care Services (2016).

## Conflict of Interest

The authors declare that the research was conducted in the absence of any commercial or financial relationships that could be construed as a potential conflict of interest.

## Publisher's Note

All claims expressed in this article are solely those of the authors and do not necessarily represent those of their affiliated organizations, or those of the publisher, the editors and the reviewers. Any product that may be evaluated in this article, or claim that may be made by its manufacturer, is not guaranteed or endorsed by the publisher.
